# Objective Assessment of Endogenous Collagen *In Vivo* during Tissue Repair by Laser Induced Fluorescence

**DOI:** 10.1371/journal.pone.0098609

**Published:** 2014-05-29

**Authors:** Vijendra Prabhu, Satish B. S. Rao, Edward Mark Fernandes, Anuradha C. K. Rao, Keerthana Prasad, Krishna K. Mahato

**Affiliations:** 1 Biophysics Unit, School of Life Sciences, Manipal University, Manipal, India; 2 Division of Radiobiology and Toxicology, School of Life Sciences, Manipal University, Manipal, India; 3 Division of Biotechnology, School of Life Sciences, Manipal University, Manipal, India; 4 Department of Pathology, Kasturba Medical College, Manipal University, Manipal, India; 5 School of Information Sciences, Manipal University, Manipal, India; University of Zurich, Switzerland

## Abstract

Collagen, a triple helical protein with the primary role of mechanical function, provides tensile strength to the skin, and plays a pivotal task in tissue repair. During tissue regeneration, collagen level increases gradually and therefore, monitoring of such changes *in vivo* by laser induced fluorescence was the main objective behind the present study. In order to accomplish this, 15 mm diameter excisional wounds were created on six to eight week old Swiss albino mice. The collagen deposition accelerated upon irradiation of single exposure of 2 J/cm^2^ He-Ne laser dose immediately after wounding was recorded by laser induced autofluorescence *in vivo* along with un-illuminated and un-wounded controls. Autofluorescence spectra were recorded for each animal of the experimental groups on 0, 5, 10, 30, 45 and 60 days post-wounding, by exciting the granulation tissue/skin with 325 nm He-Cd laser. The variations in the average collagen intensities from the granulation tissue/skin of mice were inspected as a function of age and gender. Further, the spectral findings of the collagen synthesis in wound granulation tissue/un-wounded skin tissues were validated by Picro-Sirius red- polarized light microscopy in a blinded manner through image analysis of the respective collagen birefringence. The *in vivo* autofluorescence studies have shown a significant increase in collagen synthesis in laser treated animals as compared to the un-illuminated controls. Image analysis of the collagen birefringence further authenticated the ability of autofluorescence in the objective monitoring of collagen *in vivo*. Our results clearly demonstrate the potential of laser induced autofluorescence in the monitoring of collegen synthesis during tissue regeneration, which may have clinical implications.

## Introduction

The skin being the largest organ in our body, accounts for about 16% of the net body weight and serves as a barrier against various biological, mechanical, chemical and thermal injuries [1]. Any disruption to the physiological functioning of the skin triggers a series of synchronized events leading to the corresponding healing. In other words, dermal wound healing is a well-coordinated process of restoring injured tissue structure and related biological functions [2]. The array of cell types and factors involved in the process are primarily leukocytes, lymphocytes, macrophages, fibroblasts, keratinocytes, endothelial cells, matrix metalloproteinases (MMPs), tissue inhibitor of matrix metalloproteinases (TIMPs), cytokines and growth factors. All these components work in coordination for accomplishing timely healing of the wounds [3]. The process of wound healing is broadly classified into four distinct yet interrelated phases of hemostasis, inflammation, proliferation and remodeling [4]. There have also been reports on the influence of local and systemic factors on the progression of wound healing [4]. Recently, the irradiation of wounds with visible and near infrared light (600–1000 nm) was reported to have enhanced cellular metabolism via cytochrome *c* oxidase activity, ATP synthesis [5], cellular proliferation [6], and collagen deposition [7], [8] during tissue repair and regeneration.

When the injured cells in wounds were unable to follow the orderly pattern of events, it resulted in development of complications [2] such as, wound dehiscence, ulceration, hypertrophic scars and keloid formation or even turned fatal if left untreated. This defective control mechanism is considered to be the leading cause of extended hospital stay and increased expenditure in wound management. Thus, it becomes very crucial to investigate the biochemical and morphological alterations in the wound site, enabling timely assessment and subsequent treatment plannings for early healing. In routine clinical investigations, wound size, color of the granulation tissue and odor were scored for assessing the healing of wounds. However, these examinations often fail to provide the structural information beneath the wound bed and the outcome is mostly found to be subjective [9]. There have also been reports of using the measurement of electrical impendence and tensile strength for assessing wound healing [10]. Such applications being invasive in nature, have been confined only to regular hospital practices. Conversely, the tissue histology, on the other hand regardless of its invasive nature it is still trusted as the “gold standard” methodology in finding structural modifications in wound tissue. The major concern of the clinicians is that the repetitive biopsy involved in the process of such monitoring disrupt the wound environment by creating a fresh wound each time and the consequent likelihood of infection [11]. In addition, multiple steps of tissue processing make it more time consuming thus limiting the application further. Since last two decades, research has focused on discovering non-invasive optical technologies capable of uncovering biochemical and metabolic information in-situ/*in vivo* and laser induced fluorescence (LIF) is one of them.

Laser induced fluorescence (LIF) is a sensitive optical technique which has the potential to non-invasively probe the minor biochemical changes in tissues. The key endogenous fluorophores in tissues are collagen, elastin, Nicotinamide adenine dinucleotide (NADH), keratin, melanin and hemoglobin [12] which are extensively being investigated using LIF. Collagen being the major component of the extracellular matrix plays a key role during tissue remodeling [13]–[16], which if monitored non-invasively, will provide an opportunity to assess the wound healing progression and hence will help in the planning of subsequent treatment. The prospective of second harmonic generation microscopy as a non-invasive imaging tool to monitor collagen near the wound boundaries during tissue regeneration is very well documented [17]. Likewise, Raman spectroscopy and histological techniques have also been employed to evaluate native collagen in wounds [18]. The basic purpose of adopting LIF here is to monitor collagen non-invasively during tissue regeneration by measuring the corresponding autofluorescence and testing the performance of the technique. The actual motivation for the present work came from the outcome of our previous study [19], wherein, for the first time we demonstrated the usefulness of *ex vivo* autofluorescence in assessing endogenous collagen in granulation tissue during the wound healing progression following laser therapy. In that study, the wound granulation tissue shown a gradual increase in the collagen fluorescence during all the post-wounding days along with the microscopic evidence of collagen deposition as shown by the quantitative scoring of the histological features highlighting the capability of the technique in collagen monitoring. Since collagen staining by Masson's trichome stain used in that report had limitations of being unable to differentiate between different types of collagen fibers (type I & III), and failed to differentiate between thin elastin and collagen fibers. To overcome these, we have utilized Picro-Sirius red in the present study for the identification of different collagen fibers along with the information on their occurrence and orientations. In the present study, in addition to the qualitative scoring of the Picro-Sirius red stained histological sections for thicknes, occurrence and orientation, a quantitative assessment for total collagen deposition in the tissue was also perfomed using image analysis along with its comparision with spectroscopic findings of endogenous collagen.

## Materials and Methods

### 2.1 Animal model and wound induction

Care and handling of animals was conducted in accordance with the guidelines of the World Health Organization and the Indian National Science Academy, New Delhi with prior approval from the Institutional Animal Ethics Committee (IAEC) of Manipal University, Manipal, India. Animals were kept under standard pathogen free conditions having a constant temperature (23±2°C), humidity (50±5%) and 14 and 10 h of light and dark cycles respectively. A total of 126 Swiss albino mice of six to eight week old, both sex (equal males and females) weighing 25–30 g without any signs of infections were included in the study. Animals were allowed access to sterile food and water *ad libitum*. Wound inductions on the animals were carried out under the cocktail anesthesia of Ketamine (65 mg/kg body weight) and Diazepam (8 mg/kg body weight) as per the earlier established protocol [8]. Wounds were kept open without dressing for control as well as treatment group animals throughout the experimental period. All the experimental wounds were created by the same person in an attempt to limit the variability in diameter, depth and location. Following wounding, the animals were allowed to recover, each housed in a separate polypropylene cage containing sterile paddy husk bedding.

### 2.2 Study design

#### 2.2.1 Influence of age and gender on *in vivo* autofluorescence

In this experiment, an attempt was made to investigate the influence of age and gender on the *in vivo* tissue autofluorescence. To perform this experiment a total of 36 animals were randomly selected and grouped into 3 groups of 12 animals each (six males and six females), i.e. group 1- Un-wounded control, group 2- un-illuminated control and group 3- single exposure of 2 J/cm^2^ treated animals respectively. The *in vivo* autofluorescence signals of un-wounded control animals of different age groups i.e. 7, 8, 9, 10, 11, 12, 13, 14, 15, 16 and 21 weeks were recorded. Further, to explicate the role of gender, autofluorescence signals of male and female from all the three experimental groups were recorded and compared on days 0, 5, 10, 30, 45 and 60 post-wounding.

#### 2.2.2 Monitoring of *in vivo* autofluorescence from skin/granulation tissue

This experiment was performed to investigate the predictive potential of *in vivo* autofluorescence for non-invasive collagen monitoring during excisional wound healing. A total of 45 animals were randomly distributed into 3 groups of 15 animals in each, group 1- Un-wounded control, group 2- un-illuminated control and group 3- single exposure of 2 J/cm^2^ treated animals respectively. *In vivo* autofluorescence was measured on day 0, 5, 10, 30, 45 and 60 post-wounding by exciting animal skin/granulation tissue at 325 nm and corresponding collagen intensity value with respect to normalized NADH peak for the each animal from the respective experimental groups was noted.

#### 2.2.3 Histological studies

This experiment was conducted to study the histological changes in collagen fibers (thickness, occurrence and orientation) under the influence of optimal laser dose during the progression of excisional wound healing. The animals were grouped into 3 groups of 15 animals in each i.e., group 1- Un-wounded control, group 2- un-illuminated control and group 3- single exposure of 2 J/cm^2^ treated animals respectively. A total of 45 animals was utilized for this experiment. The granulation tissue/skin samples from 3 animals of each experimental group was excised following euthanization on days 5, 10, 30, 45 and 60 post-wounding, and were fixed in Bouin's fixative (24 h) for further histological analysis.

Skin/granulation tissues were subjected to a series of dehydration with graded alcohol, cleared in xylene and embedded in paraffin wax. 5–7 µm thick tissue sections were taken manually using Leica rotary microtome. Subsequently, a pair of slides from each animal was stained with Picro-Sirius red as per the earlier reported protocols [20]. Tissue sections were stained with 0.1% Picro-Sirius red and the birefringence patterns for the same were visualized with a conventional optical microscope fitted with polarizing filters. From each slide colour images were captured using light microscope with digital camera running under image analysis programme. Further, Picro-Sirius stained slides from each animal of each experimental group were qualitatively assessed in a blinded manner for different parameters related to color, size, orientation and occurrence of collagen fibers as listed in Table 1. Furthermore, image analysis of the histological sections were also performed.

**Table 1:** Scoring parameters for the collagen assessment in histological sections.

Sl No	Feature	Types	Occurence	
			Presence	Absence
1	Fiber color	Yellow	+	_
		Red	+	_
		Green	+	_
2	Size of fibers	Thick	+	_
		Thin	+	_
3	Orientation of fibers	Horizontal	+	_
		Vertical	+	_
4	Occurrence in the dermal layers	Papillary dermis	+	_
		Mid dermis	+	_
		Deep dermis	+	_

### 2.3 Light source and treatment procedure

The light source utilized in the present study was described in detail elsewhere [8]. The animals assigned to laser treatment group were illuminated with the single exposures of He-Ne laser (632.8 nm; 7 mW; 4.02 mW/cm^2^) at 2 J/cm^2^ (8 min 32 sec) immediately following wounding, while un-illuminated control animals were not given any irradiation. In brief, the red laser beam was focused to an optical fiber (200 µm- core), which was coupled to a beam expander (Expansion ratio: 2.5X-15X) held vertically at a separation of 20 mm from the wound site making non-contact mode of laser irradiation. All the procedures related to animal handling and wounding were identical to rule out the possible influence of the above factors on the outcomes of the experiment.

### 2.4 Autofluorescence signal recording

**Figure 1:**
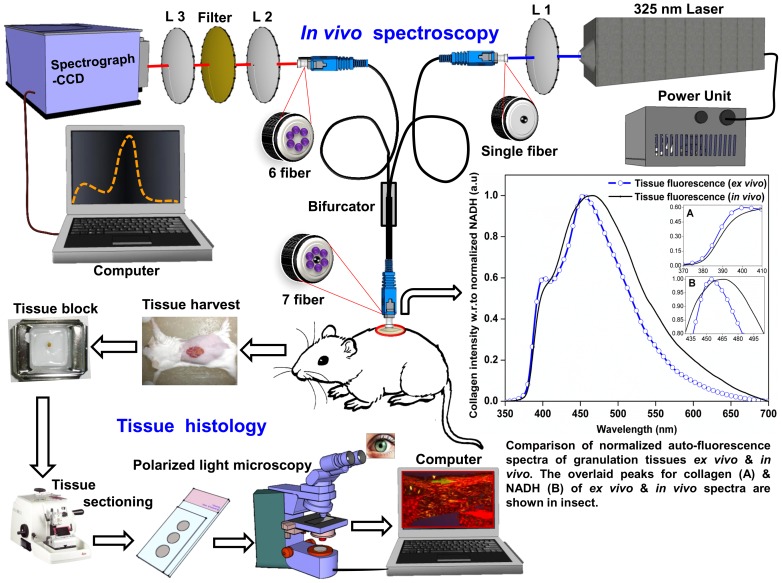
Experimental design. Schematic layout of the experimental setup for recording *in vivo* autofluorescence and microscopic studies. Typical overlaid *ex vivo* and *in vivo* autofluorescence spectra of granulation tissue at 325 nm excitation with peaks for collagen (A) & NADH (B) shown in the inset.

The schematic layout of the experimental setup used to record *in vivo* autofluorescence in the present study is shown in Figure 1. The details of the experimental setup is explained elsewhere [19]. In brief, the 325 nm laser excitation was focused onto the single fiber (central excitation and six collection fibers) of the probe using focusing lens (L1), to carry the light to the target site under study. The probe carrying laser light was mounted perpendicular (90°) to the measurement site, with a stable power of 2 mW at a constant separation of 3 mm (from the tip of the fiber probe to the tissue surface) making it non-contact mode of spectral recording. Also, the delivered laser power to the tissue site during the autofluorescence recordings were well within the maximum permissible exposure guidelines defined by American National Standard Institute [21].The micro spectrometer detector having 2-D arrangements of pixels 1044×64, with a responsive range 200–1100 nm was utilized to record the spectrum. All the spectra were recorded with a 5 second integration time.

The *in vivo* autofluorescence spectra were recorded from all the experimental group animals on day 0, 5, 10, 30, 45 and 60 post-wounding. Prior to spectral recording, all the animals of the respective experimental groups were anesthetized under the cocktail anesthesia of Ketamine (65 mg/kg body weight) and Diazepam (8 mg/kg body weight). For spectral measurements, four different sites of the wounded tissue/skin were selected and four spectra were recorded from each site. An average spectrum was then computed of all the recorded spectra from a site and was used for further analysis.

### 2.5 Spectral processing

In the spectroscopic measurements, 16 spectra were recorded for each animal from 4 different sites of a skin/granulation tissue and averaged for further analysis. To study the influence of gender and age on *in vivo* autofluorescence properties, a total of 2880 spectra was recorded from 36 animals. For monitoring *in vivo* autofluorescence, a total of 4320 spectra was recorded from 45 animals. All the spectra were smoothed, baseline corrected and normalized using GRAMS/AI 8.0 spectroscopy software. In the present study, Fourier smoothing was accomplished by Fourier transforming the data, applying a filter function and then doing an inverse Fourier transform on that. For all the spectra 80% smoothing was applied. Generally, the spectrometer does not generate spectrum with an ideal baseline which may not be suitable for normalization. Therefore, baseline correction is an essential step in spectral pre-processing. In the present study, two point baseline corrections (selecting start and end point of the spectrum) was used. Following smoothing and baseline corrections, normalization of the spectra were done. The spectra were normalized corresponding to I_max_. Since all the spectra had two major peaks i.e. Collagen at ≈405 nm; NADH at ≈455 nm being the highest peak intensity, all the spectra were normalized with respect to NADH intensity. Once all the spectra were normalized, the peak intensity corresponding to 405 nm representing collagen was computed. The average of the same was then represented as collagen intensity for all the experimental group and time points under study. Subsequently, the area under the curves (AUC) for collagen peaks in the spectral range 350–405 nm were calculated by numerical integration using the Microcal Origin 8.6 software. The averaged AUC values of each experimental group at different post-wounding days were compared to ascertain the collagen levels.

### 2.6 Image analysis

Digital images of Picro-Sirius red stained histological slides were acquired at 10 X-magnification and the corresponding image analysis was performed using the “TissueQuant” software, specially developed for quantification of color intensities [22]. The software provides a choice for color selection and their shades in the positively stained areas of an image. The type I collagen fibers which were observed bright red/yellow in color and its shades in the image were selected after adjusting the color settings in the software. Similarly, type III collagen fibers with weak green birefringence were also adjusted for green color and its shades in the image. Once the color settings were corrected for the true color and its corresponding shades, these reference settings were then used for further analysis. The software automatically calculates and provides scores to each of the selected colors (red, yellow, green), representing total quantity of the type of collagen present in the captured field. The Scores refer to intensity of given colors in each pixel. Through this software, it is possible to calculate the “area” (of each pixel) as well as the “score” representing the corresponding color intensity. Color shades closer to the reference color are given high scores, distant shades with lower scores and color shades other than user specified reference color are given zero score. Average and standard error of the mean was computed for laser treatment and un-illuminated control groups on all of the time points studied. Further, total amount of collagen deposited was represented as percentage scores in un-illuminated control and test groups by adding the scores of type I (red and yellow) and type III (green) collagen together for comparing it with the corresponding spectroscopic findings.

### 2.7 Statistical analysis

All the data were expressed as mean ± SEM. For spectroscopic studies, statistical significance among the un-illuminated control and the laser treatment group was determined using repeated measures ANOVA with Bonferroni's post hoc test. Comparisons of total collagen values among the experimental groups were performed by the Student's unpaired two tailed *t*-test. For statistical analysis GraphPAD Prism- 5 software was used. Values of *P*<0.05 were considered significant.

## Results

### 3.1 Comparison of the *ex vivo* and *in vivo* autofluorescence spectral profiles

The overlaid *ex vivo* and *in vivo* normalized tissue autofluorescence spectra due to the endogenous collagen and NADH as shown in figure 1 are almost similar, only showing a slight red shift in the emission maxima for *in vivo* as compared to the *ex vivo* spectral patterns at 400 nm and 455 nm respectively.

### 3.2 Influence of age and gender on *in vivo* autofluorescence

**Figure 2:**
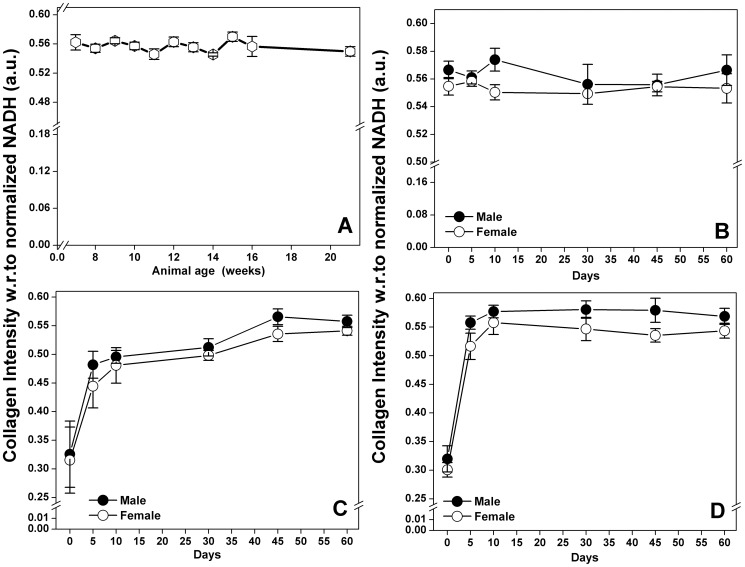
Autofluorescence patterns with varying age and gender. Influence of age on autofluorescence pattern of collagen in un-wounded skin (A). Average collagen intensity was plotted against different age group (in weeks). Collagen intensity values with respect to normalized NADH levels for male and female animals of un-wounded control (B), un-illuminated control (C), and optimum laser dose treated group (D) on different post-wounding days.

The influence of age on the *in vivo* collagen autofluorescence from un-wounded skin of 7, 8, 9, 10, 11, 12, 13, 14, 15, 16 and 21 week old Swiss albino mice was inspected and the endogenous collagen levels in them were computed. The variations in the collagen intensity with the increase in age (in weeks) is plotted as shown in Figure 2A. The collagen intensity with respect to normalized NADH intensity were ranged from 0.5454 to 0.5698 (a.u). The lowest collagen intensity of 0.5454 (a.u) was recorded for 14 week old animals, whereas the highest collagen intensity of 0.5698 (a.u) was recorded for 15 week animals. The observed change in the endogenous collagen intensity could be attributed to age, however this minute variation in collagen intensity with respect to age was not found to be statistically significant during the experimental period of the study.

Collagen content from the un-wounded control (Figure 2B), un-illuminated control (Figure 2C) and laser treated animals (Figure 2D) were recorded *in vivo* at different time points (0, 5, 10, 30, 45 and 60 days post-wounding). Collagen signals from the un-wounded skin at different time points were served as controls providing information about the collagen levels in the normal intact skin. Male skin collagen content in un-wounded animals (Figure 2B) was ranging from 0.5556±0.0144 to 0.5738±0.0082 (a.u), as compared to the female skin collagen levels of 0.5493±0.0076 to 0.5582±0.0036 (a.u) respectively. It could be noticed that the collagen content in male un-wounded skin was found to be higher compared to the female skin in all the recorded time points, however, this difference between the gender (within the group) was not found to be statistically significant. Similar trends have also been observed in un-illuminated control and laser treated animals. The baseline values for average collagen intensities in males and females for un-illuminated controls and laser treatment group was compared but the difference was not found to be statistically significant (Figure 2C, 2D). In un-illuminated group (Figure 2C), collagen intensity range in males (0.3257±0.0577 - 0.5656±0.0139 a.u) and females (0.3152±0.0577- 0.5411±0.0077 a.u) showed a progressive increase from day 0 to day 60 post-wounding indicating the healing progression. Likewise, in the optimum laser treated group (Figure 2D), average collagen intensity values of 0.3189±0.0229, 0.5578±0.0114, 0.5771±0.0109, 0.5806±0.0152, 0.5794±0.0211 and 0.5688±0.0140 a.u was recorded for male granulation tissue on days 0, 5, 10, 30, 45 and 60 post-wounding respectively. Similarly, average collagen intensity values of 0.3007±0.0125, 0.5165±0.0229, 0.5577±0.0206, 0.5467±0.0203, 0.5356±0.0118 and 0.5436±0.0130 a.u were observed in female granulation tissue on day 0, 5, 10, 30, 45 & 60 post-wounding respectively. In the animals which had undegone opptimum laser dose trreatment, highest collagen intensity in males and females was witnessed on day 30 (0.5806±0.0152) and day 10 (0.5577±0.0206) post-wounding respectively. Identical to the un-wounded control animals, the collagen deposition was found higher in males compared to the female counterparts of both un-illuminated controls and optimum laser treatment groups in all the tested time points, yet the difference was not found to be statistically significant.

### 3.3 Monitoring of *in vivo* autofluorescence from skin/granulation tissue

**Figure 3:**
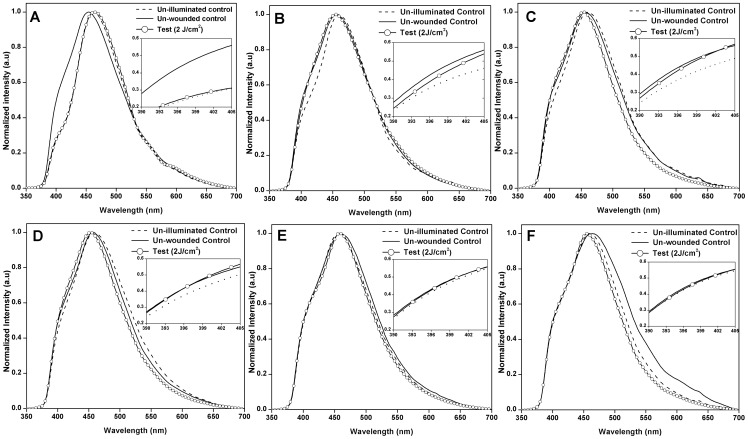
Typical *in vivo* autofluorescence spectra. Typical normalized *in vivo* autofluorescence spectra of un-illuminated control, unwounded control and 2 J/cm^2^ treated animals on day 0 (A), day 5 (B), day 10 (C), day 30 (D), day 45(E), and day 60 (F) post-wounding. Inset- Comparision of the spectral range 390–405 nm indicating the collagen peak of the all the experimental group animals.

The typical autofluorescence spectrum of the skin/granulation tissue of different experimental groups obtained *in vivo* at post-wounding days 0, 5, 10, 30, 45 and 60 were shown in figure 3 (A–F) respectively. All the denoted spectra were normalized with respect to their highest peak intensity. Progressive increase in the collagen intensity values with respect to normalized NADH levels were observed in un-illuminated control and laser treatment group on days 5, 10 and 30 (figure 3 B, C and D) post-wounding respectively. It was evident that a single exposure of optimum laser dose (2 J/cm^2^) increased the collagen content on days 5, 10 and 30 post-wounding compared to un-illuminated control animals as reflected by the *in vivo* autofluorescence spectra. On days 45 and 60 (Figure 3 E, and F) post-wounding, there was only negligible change observed in collagen intensity among the experimental groups.

**Figure 4:**
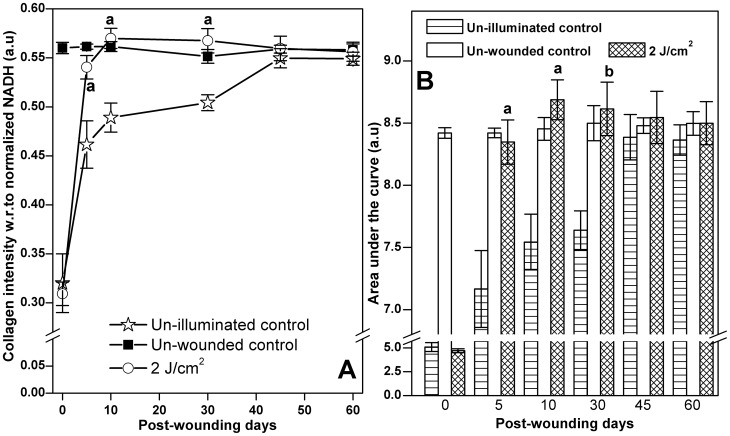
Collagen intensity and the corresponding area under the curve (AUC) values from *in vivo* granulation tissue/skin autofluorescence. Collagen intensity with respect to normalized NADH values on days 0, 5, 10, 30, 45 and 60 post-wounding for un-wounded control, un-illuminated control and optimum laser treated animals indicating the increased levels of collagen in the laser treated group (A). The AUC values for un-wounded control, un-illuminated control and optimum laser treated animals were also indicating the elevated levels of collagen in the test group (B). Level of significance ^b^ *P*<0.01, ^a^ *P*<0.001 and no symbol =  insignificant compared to un-illuminated control.

Average collagen intensity values with respect to normalized NADH levels for each experimental group on days 0, 5, 10, 30, 45 & 60 post-wounding was shown in Figure 4 A. The un-wounded intact skin providing the information on collagen levels at different experimental time points served as normal control. Collagen intensity values of 0.5600±0.0058, 0.5614±0.0026, 0.5614±0.0050, 0.5514±0.0070, 0.5589±0.0040 and 0.5580±0.0061 a.u was observed for days 0, 5, 10, 30, 45 & 60 post-wounding respectively for un-wounded controls. As no wound was created in this group of animals, the collagen intensity values with respect to normalized NADH were found relatively stable. Average collagen intensities of un-illuminated control and laser treatment group animals at different time intervals were compared with the un-wounded animals to understand the progression of healing. Collagen intensity values of 0.3200±0.299 and 0.30919±0.1204 a.u was recorded for un-illuminated control and laser treatment group of animals on day 0 indicating the baseline value for collagen immediately following wounding. Average collagen intensity values for the un-illuminated control group animals were gradually increased in all the time points from 5^th^ day to 45^th^ day post-wounding trying to attain the normal collagen levels as that off un-wounded skin. The intensity values of 0.4615±0.0241, 0.4890±0.0148, 0.5042±0.0081, 0.5496±0.0097 & 0.54909±0.0066 a.u was noted on days 5, 10, 30, 45 & 60 post-wounding for un-illuminated control animals. Single exposure of optimum laser dose (2 J/cm^2^) had a positive influence on the synthesis of collagen which was reflected in the *in vivo* autofluorescence readings of collagen intensities with values of 0.5404±0.012, 05698±0.0103, 0.5675±0.0123 a.u on days 5, 10 & 30 post-wounding. As a result of extensive collagen remodeling on days 45 & 60 post-wounding, intensity values (0.5596±0.0124 & 0.5562±0. 0096 a.u.) for these time points were found much stabilized in the laser treatment groups. In all the post-wounding days (5, 10, 30, 45, 60), the endogenous collagen spectral intensity was higher in the laser treatment animals as compared to the un-illuminated control group. This difference in the collagen intensity in the optimum laser treatment group as compared to un-illuminated control animals on day 5, day 10 and day 30 post-wounding was found to be statistically significant (*P*<0.001). In contrast, on days 45 and 60 post-wounding, the collagen intensity values in both un-illuminated control and laser treatment group were comparable to the collagen intensity of un-wounded skin.

Besides the spectral intensity, one more spectral parameter, the area under the curve (AUC) of the collagen peak (350–405 nm) was also used to support the spectral intensity measurments at 405 nm in all the spectra. The AUC of the collagen peaks were computed for the respective experimental groups on all of the time points and an average value for the same were used for the comparison. The AUC values for the un-wounded control, un-illuminated control and the optimum laser treated animals were plotted against different time points as shown in Figure 4B. The AUC of collagen peak values of 8.4200±0.0425, 8.4212±0.03852, 8.4535±0.0913, 8.4994±0.1408, 8.4798±0.0623 & 8.3642±0.1214 a.u was obtained on days 0, 5, 10, 30, 45 & 60 post-wounding indicating the stable collagen levels in the intact un-wounded skin throughout the observational period. Likewise, the collagen spectral intensity, the AUC for the collagen peaks were increasing progressively in both the un-illuminated control and laser treatment groups from day 0 to day 30 post-wounding, following which it was stabilized on day 45 and day 60 post-wounding respectively. The AUC values for the collagen peaks in the laser treated animals on day 5, 10, 30, 45 & 60 post-wounding were found higher than un-illuminated control, and this difference in the AUC values were also found statistically significant (*P<0.001* on days 5 & 10; *P<0.01* on days 30) as compared to the un-illuminated controls.

### 3.4 Histological studies of granulation tissue

**Figure 5:**
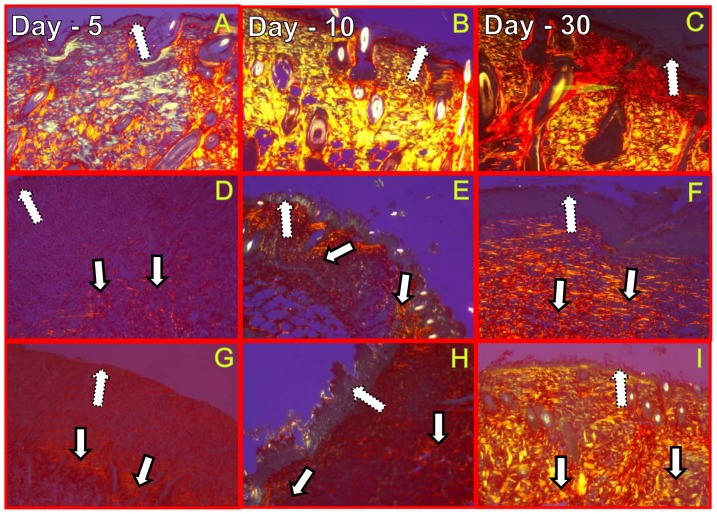
Collagen birefringence under polarized light microscopy. Histological sections of the un-wounded control, un-illuminated control, and 2 J/cm^2^ treatment group on day 5, 10, 30 post-wounding stained with a direct red visualized by the polarized microscope (10X). Collagen birefringence pattern of un-wounded skin (A, B, C,), Un-illuminated control (D, E, F) and optimal laser treated group (G, H, I) on days 5, 10, 30 post-wounding respectively. A skin section with hair follicles (A, B, C), Loosely formed early collagen (D), moderate collagen (E), birefringent yellow/red fibers (F), Moderately birefringent red/yellow fibers (G), thick strongly birefringent yellow/red fibers (H) and strongly birefringent thick red fibers (I) as indicated by arrows. Dotted arrow indicates epidemis/epidermal layer and solid arrows indicate the occurrence of collagen.

Histological sections of all the three experimental groups on day 5, day 10 and day 30 post-wounding were stained with Picro-Sirius red as shown in figure 5. As expected, histological sections of the un-wounded intact skin were alike on all the three days (5, 10 and 30) without displaying any structural changes. Progressive increase in the collagen deposition was exhibited (Figure 5) from the histological sections of the granulation tissue/regenerated skin of un-illuminated control and optimum laser treated group on days 5, 10 and 30 post-wounding. Exposure to optimum laser dose enhanced the collagen deposition in terms of thickness and distribution of the fibers throughout the regenerated tissue as shown in the figure 5.

Histological sections were examined in a blinded fashion and scored qualitatively for the thickness of the collagen fiber, their occurrence and orientations by an expert pathologist. The scores for different histological parameters of un-wounded control, un-illuminated control and laser treatment groups were listed separately in tables 2, 3 and 4 respectively. As there was no wound created in the un-wounded control group of animals, the scores for colour, size, occurrence and orientation of collagen fibers were relatively found to be similar on days 5, 10, 30, 45 and 60 post-wounding respectively as shown in Table 2. The brightly birefringent red and yellow, thin and thick fibers were evenly distributed in mid and deep dermis of the skin (day 5, day 10, day 30, day 45 and day 60) as visualized using plane polarized light. Most of the collagen fibers were horizontally oriented (parallel to the plane) except on day 10, where the thick red and yellow birefringent fibers were found oriented vertically. On day 5 and day 30 un-wounded skin, the occurrence of fibers was seen even in the papillary dermis in addition to the mid and deep dermis.

**Table 2:** Qualitative scoring for the collagen assessment in histological sections of Un-wounded control.

Group	Day	Fiber colour	Size		Distribution in Dermal layers			Orientation	
			Th	Tn	P	M	D	H	V
Un-wounded control	5	Yellow	+	_	+	+	_	+	_
		Red	+	+	+	+	+	+	_
		Green	_	_	_	_	_	_	_
	10	Yellow	+	_	_	+	+	_	+
		Red	+	_	_	+	+	+	+
		Green	_	_	_	_	_	_	_
	30	Yellow	+	_	+	+	+	+	_
		Red	+	+	+	+	+	+	_
		Green	_	_	_	_	_	_	_
	45	Yellow	_	_	_	_	_	_	_
		Red	+	_	_	+	+	+	_
		Green	_	_	_	_	_	_	_
	60	Yellow	+	_	_	+	+	+	_
		Red	_	+	_	+	+	+	_
		Green	_	_	_	_	_	_	_

Note.

Size of the fiber: Th- Thick; Tn-Thin;

Dermal layers: P- Papillary; M- Mid; D- Deep;

Orientation of the collagen fiber: H- Horizontal; V- Vertical.

**Table 3:** Qualitative scoring for the collagen assessment in histological sections of Un-illuminated control.

Group	Day	Fiber colour	Size		Distribution in Dermal layers			Orientation	
			Th	Tn	P	M	D	H	V
Un-illuminated control	5	Yellow	_	_	_	_	_	_	_
		Red	_	_	_	_	_	_	_
		Green	_	+	+	_	_	+	_
	10	Yellow	_	+	+	_	_	+	_
		Red	_	_	_	_	_	_	_
		Green	_	_	_	_	_	_	_
	30	Yellow	+	+	+	+	_	+	+
		Red	_	_	_	_	_	_	_
		Green	_	_	_	_	_	_	_
	45	Yellow	_	_	_	_	_	_	_
		Red	+	_	_	+	+	+	+
		Green	_	_	_	_	_	_	_
	60	Yellow	_	_	_	_	_	_	_
		Red	+	_	+	+	+	+	+
		Green	_	_	_	_	_	_	_

Note.

Size of the fiber: Th- Thick; Tn-Thin;

Dermal layers: P- Papillary; M- Mid; D- Deep;

Orientation of the collagen fiber: H- Horizontal; V- Vertical.

**Table 4:** Qualitative scoring for the collagen assessment in histological sections of optimum laser treated group.

Group	Day	Fiber colour	Size		Distribution in Dermal layers			Orientation	
			Th	Tn	P	M	D	H	V
Laser treatment (2 J/cm^2^)	5	Yellow	+	+	+	+	_	+	_
		Red	_	_	_	_	_	_	_
		Green	_	_	_	_	_	_	_
	10	Yellow	_	_	_	_	_	_	_
		Red	+	_	+	+	_	+	+
		Green	_	_	_	_	_	_	_
	30	Yellow	+	_	+	+		+	_
		Red	+	_	_	+	+	+	_
		Green	_	_	_	_	_	_	_
	45	Yellow	_	_	_	_	_	_	_
		Red	+	+		+	+	+	_
		Green	_	_	_	_	_	_	_
	60	Yellow	_	_	_	_	_	_	_
		Red	+	_	_	+	+	+	_
		Green	_	_	_	_	_	_	_

Note.

Size of the fiber: Th- Thick; Tn-Thin;

Dermal layers: P- Papillary; M- Mid; D- Deep;

Orientation of the collagen fiber: H- Horizontal; V- Vertical.

Scores of Picro-Sirus red stained granulation tissue of un-illuminated control (Table 3) on day 5 indicated the presence of weakly birefringent green fibers. These horizontally oriented thin fibers were localized only in the papillary dermis of the tissue. On day 10 and day 30, the green fibers were replaced by strong birefringent yellow fibers. On day 10 post-wounding, horizontally oriented thin yellow birefringent fibers were distributed evenly in the papillary dermis. However, on day 30 post-wounding, both thick and thin fibers were viewed and distribution of these fibers was extended upto mid dermis of the regenerated skin. Both types of fiber orientation (horizontal and vertical) were seen on day 30 post-wounding in the un-illuminated control group of animals. Further, on day 45 and day 60 post-wounding, thick bright red birefringent fibers were seen dispersed till deep dermis, with both horizontal and vertical orientations. Single exposure of the optimum laser dose had an affirmative effect on collagen deposition during the early granulation tissue formation which was revealed by qualitative scoring (Table 4) of the laser treated granulation tissue/regenerated skin on different time points. Mixture of thin and thick yellow birefringent fibers were seen early on day 5 post-wounding, distributed in the papillary and mid dermis of the newly synthesized granulation tissue. On day 10 post-wound irradiation, more prominent bright red birefringent collagen fibers were seen distributed in papillary and mid dermis in both horizontal and vertical orientations. Mixture of thick bright red/yellow collagen fibers, diffused throughout the mid and deep dermis was seen on day 30 post-wound irradiation. Similarly, laser treated group animals on day 45 and day 60 post-wound irradiation exhibited horizontally arranged red birefringent thick collagen fibers distributed in the mid and deep dermis. On comparing the scores of the histological sections of un-illuminated controls (Table 3) and optimum laser treated groups (Table 4), it was evident that laser exposure had played a major role in the synthesis of collagen fibers in terms of type, size, occurrence and the orientation of the fibers during the progression of wound healing.

**Figure 6:**
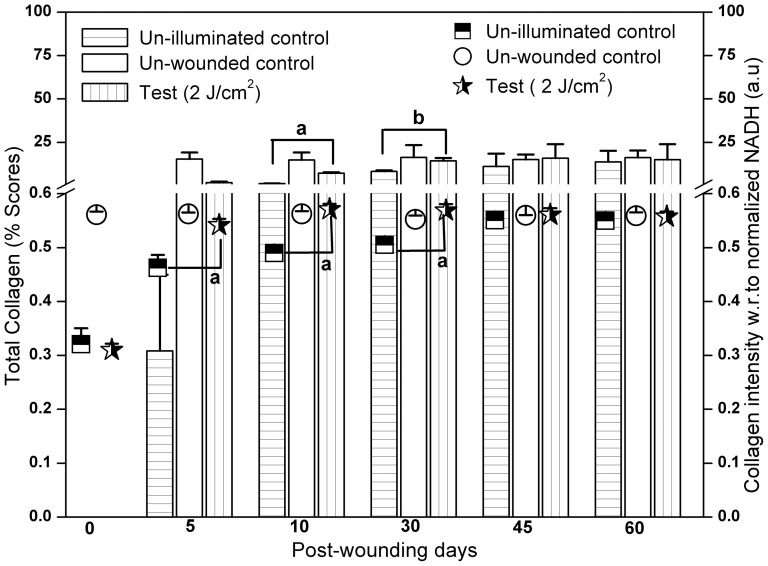
Comparison of the change in collagen levels measured by autofluorescence and polarized microscopy (image analysis). Bar graph indicates the total collagen (% scores) derived from image analysis of Picro-Sirius red stained histological sections. Total amount of collagen for each group was computed by adding the type I (yellow and red fibers) and type III (green fibers) together. Scatter plot indicates collagen intensity with respect to normalized NADH levels. Results were represented as mean ±SEM. Level of significance ^b^ *P*<0.05, ^a^ *P*<0.001 and no symbol =  nonsignificant compared to un-illuminated control.

Further to compare the spectral findings with the Picro-Sirius red stained histological sections on collagen deposition, image analysis was performed to get a quantitative assessment between them. Comparison of the change in collagen levels measured by autofluorescence (bar plot) and polarized microscopy (scatter plot) was shown in figure 6. The total amount of collagen for un-wounded skin did not alter greatly as the percentage score values of 15.33±3.83, 14.77±4.34, 16.21±7.15, 15.01±2.92 and 16.18±4.09 on days 5, 10, 30, 45 and 60 post-wounding respectively. In un-illuminated control the total collagen values of 0.30±0.14%, 1.21±0.02%, 8.24±0.54%, 11.06±7.34% and 13.67±6.37% were recorded on 5, 10, 30, 45 and 60 days post-wounding respectively. Single exposure of optimum laser dose markedly elevated the total collagen content with scores of 1.76±0.56%, 7.16±0.51%, 14.28±1.67%, 15.88±7.98% and 14.98±8.89% on days 5, 10, 30, 45 and 60 post-wounding. This laser assisted collagen deposition was found to be significant on day 10 (*P*<0.001) and day 30 (*P*<0.05) post-wounding. However, the difference was not found to be significant on day 5 (*P* = 0.057), day 45 (*P* = 0.679) and day 60 (*P* = 0.910) post-wounding as compared to the un-illuminated controls. Conversly, the increase in the collagen intensity in laser treated animals measured by *in vivo* autofluorescence on day 5, day 10 and day 30 post-wounding was found to be significant (*P*<0.001) compared to un-illuminated controls.

## Discussion

Presently available methods of examining collagen in biological tissues are histochemistry and immunohistochemistry wherein both techniques involve several steps in tissue processing and it introduce undesirable structural variations in the collagenous matrix [14]. Other techniques used to monitor collagen include high resolution ultrasound, thermography, laser doppler imaging, polarization imaging, fluorescence imaging [23], small angle X ray imaging [24], magnetic resonance imaging [25], confocal microscopy [26], electron microscopy [27] etc. These imaging techniques most often experience low chemical specificity and low spatial resolution [14]. Earlier in this line, in order to overcome these limitations we had monitored collagen autofluorescence *ex vivo* during tissue regeneration followed by laser therapy [19] at different post-wounding days (5, 10 and 15) supported by histological examination with quantitative scoring in a blinded fashion. In addition to collagen deposition, six different histological parameters (edema, leukocytes, macrophages, fibroblast, granulation tissue and epithelialization) related to different phases of wound healing have also been quantitavely scored by Haematoxylin –eosin/Masson's trichome staining. The results obtained have clearly indicated the potential of laser induced autofluorescence in collagen monitoring during wound healing progression. Thus, in the present study our idea was to extend the applicability of LIF further for objective and non-invasive assessment of collagen during tissue regeneration in a pre-clinical model.

It is a well-known fact that metabolic and biochemical properties of tissues vary significantly during *in vivo* and *ex vivo* assessments [28]. This could be attributed to the variations in NAD^+^/NADH ratio, changes in the oxidation state and the blood contents. Further, the autofluorescence spectral patterns of tissues are largely dependent on these alterations as well, reflecting the change in the corresponding spectra [29]. The present study on the other hand exhibited almost similar autofluorescence as that of *ex vivo* with a minor shift in the corresponding collagen and NADH emission, suggesting its possibile *in vivo* application as a real time monitoring of collagen. It has been reported that *in vitro*/*in vivo* studies can introduce a significant artifacts in the spectral patterns due to hemoglobin reabsorption [29]. In our previous report [19], it was observed that *ex vivo* autofluorescence spectra had a more prominent collagen peak at 400 nm as compared to *in vivo* autofluorescence. This could be attributed to the hemoglobin reabsorption in *ex vivo* conditions showing a dip at around 420 nm, there by making collagen peak (hump) at 400 nm more prominent. In the present study since, no attempt was made to assess the exact contribution of hemoglobin reabsorption; its influence on the recorded *in vivo* spectral patterns can't be ignored. Having said that, it would be advisable to factor-in the contribution of hemoglobin reabsorption before advocating the use of autofluorescence technique in clinical settings.

The mouse models have comprehensively been explored over the years to inspect diverse skin diseases and tumor growths through optical measurements including photodynamic therapy [30], *in vivo* bioluminescence fluorescence imaging [31] etc. In the present study, an attempt has been made to understand the variations in the endogenous collagen signatures with varying age and gender. To this end, corresponding autofluorescence spectra were recorded *in vivo* on un-wounded skin of Swiss albino mice of age ranging from 7–21 weeks. The pioneering work on the influence of age and sex on collagen autofluorescence was reported earlier by Shuster and co-workers [32] involving human subjects which concluded that skin collagen decreases with age. On the contrary, in a study by Kollias and co-workers [33], a positive correlation between age and collagen deposition by measuring endogenous skin fluorescence using albino hairless mouse models was observed. In the present study although, there were variations in the collagen intensities among un-illuminated control and laser treatment groups with age, it was found to be nonsignificant, may be attributed to the selected age range (7–21 weeks), which would have been improved if the age range selected was between 5–55 weeks. Similarly, autofluorescence results indicated higher collagen deposition in male mice compared to females in all the tested time points, however the difference in collgen deposition was not found to be statistically significant. Present report is the first of its kind, wherein autofluorescence technique has been utilized to monitor collagen non-invasively as well as to reveal the differential collagen deposition with gender during acute wound healing.

Previous studies from our laboratory contributed significantly in demonstrating the augmented effect of red light laser irradiation on collagen synthesis in pre-clinical models during tissue regeneration [7], [8], [19]. Thus, the major focus of the current work was to elucidate the prognostic potential of *in vivo* autofluorescence in the factual measurement of collagen rather than proving the beneficial effect of LLLT in collagen deposition (which has already been proved earlier). In order to achieve the stated objective, normalized spectral intensity and area under the curves for collagen peaks were computed and compared. In our previous report [19], we have utilized normalized NADH/collagen ratio to assess the collagen deposition in the spectra. However, in the present study we believed that the inclusion of collagen intensity and an additional parameter such as AUC could help in acquiring more accurate information about the collagen. Although, collagen intensities and AUC were extracted from the same spectra, we believe that the intensity could provide information at a single point i.e., at a wavelength of 405 nm whereas, AUC could provide supplementary information to the spectral intensity values. Thus, AUC measurments in the present study were performed only to support/confirm the findings of collagen intensity at 405 nm. Single exposure of the optimum laser treated animals displayed 1.171, 1.165, 1.125, 1.020 and 1.012 fold increase in collagen intensity (Figure 4A) and 1.165, 1.151, 1.127, 1.019 and 1.016 fold increase in AUC values (Figure 4B) compared to un-illuminated controls on days 5, 10, 30, 45 and 60 post-wounding respectively. Two different spectral parameters i.e. spectral intensity and AUC displayed similar and comparable value for collagen deposition substantiating the potential of *in vivo* autofluorescence technique in investigating collagen deposition during the progression of healing. Our findings on collagen monitoring at different post-wounding days was similar to the previously published reports [34] hypothesizing that the *in vivo* autofluorescence is equally efficient in providing the unprejudiced information on collagen deposition as compared to the available techniques for the same. In the present study, though spectral shape was not prominent among the experiment groups as compared to the *ex vivo* autofluorescence spectral patterns, the analysis pertaining to collagen intensity and AUC was successful in reflecting the minute changes in the collagen levels during the healing progression.

A review of latest work published by Deka and co-workers [35], [36] have shown a higher metabolic rate (measured by free to bound NADH ratio) during inflammation and initial cell migration stages of wound healing [35], whereas a reduced cellular metabolic activity was observed during the tissue remodeling stage after day 10 post-wounding [36]. In the same study, lower collagen signals were recorded till day 4 post-wounding, followed by increased collagen levels (after 5^th^ day) till day 20 post-wounding as measured by polarization resolved second harmonic imaging. These latest reports thus suggests that, the monitoring of NADH and collagen can serve as ideal markers for non-invasive assessment of wound healing progression and their regulation was not dependent on each other during the progression of tissue repair. Since the NADH levels vary during the wound healing progression, spectral normalization with NADH levels employed in the present study might have some limitations in the measurement.

The collagen of present interest, type I and III belong to the classical fibril forming collagens, constitute more than 90% of the skin collagen content. The depleted or excessive accumulation of this extracellular matrix component serves as a marker for the numerous pathological conditions including cutaneous collagenoma, dermal fibrosis, hypertrophic scars and keloids [37]. Traditional morphometric methods such as hematoxylin and eosin [38], van Gieson, Masson's trichome and immunohistochemical detection were routinely employed to detect collagens in diverse pathological conditions [39]. However, hematoxylin and eosin lacks the specificity for collagen [38], whereas van Gieson and Masson's trichome failed to stain very thin collagen fibers leading to the under representation of the collagen content [39]. The non-availability of the specific antibodies in a few animal models and the cost involved for the same serves as a major limitation for the utilization of immunohistochemical techniques for determining collagen content [40]. This led to the discovery of a simple, sensitive, reliable and cost effective Picro-Sirius red- polarization method to differentiate between the different collagen types in tissues [41]. In addition, by utilizing polarized light microscopy, it provides vital informations on fiber orientation, thickness and distribution in the tissues. Hence, Picro-Sirius red-polarization method has widely been used to examine the collagen orientation and distribution in various studies [34], [40]–[42] including ours.

Microscopic examinations of the histological sections of un-wounded skin indicated the minimal changes in the collagen morphology in terms of collagen type, size and distribution and orientations. In all the experimental days (5, 10, 30, 45 and 60 post-wounding) bright red/yellow birefringent fibers visualized (Table 2). Vertically oriented red/yellow birefringent fibers were seen in un-wounded skin on day 10. Similar findings of occurrence of randomly oriented collagen fibers have also been reported from the earlier studies as a way to resist different mechanical/shearing forces [43]. The microscopic images of the histological sections of un-illuminated control on days 5 and 10 post-wounding indicated the presence of thin weakly birefringent green and yellow fibers localized in papillary dermis of the regenerating tissue (Table 3). On day 30 post-wounding, localization of yellow birefringent fibers were seen till mid dermis indicating the progression of healing. However, on day 45 and 60 post-wounding, yellow fibers were replaced by more bright red birefringent type I collagen. More interestingly, newly formed collagen fibers were vertically oriented in addition to parallel orientations. An exact explanation for the occurrence of vertically oriented collagen fibers in the regenerated skin is not clearly known. Single exposure of the optimum laser dose had significantly influenced the collagen content which was evident by the occurrence of both thick and thin fibers in papillary and mid dermal layers even on day 5 post-wounding (Table 4). In addition, brightly birefringent red fibers was prominently present in histological sections of the optimum laser treated animals on days 10, 30, 45 and 60 post-wounding clearly indicating the simulative effect of visible red light on collagen synthesis. On day 30 post-wounding, the type I collagen localized mainly in mid and deep dermal layers of the regenerated skin indicating the succession of healing under the influence of He-Ne laser.

Further, image analysis was performed for histological sections to quantify the total collagen deposited during the progression of healing. This was necessitated by the fact that histological assessments (mentioned above) were qualitative and the prognostic potential of *in vivo* autofluorescence could only be tested with quantitative assessments through gold standard measurments. In the present study, since spectral discrimination of type I and III collagen were not performed, the results of image analysis of the histological sections were represented as total collagen (sum of green fiber (type III), yellow and red fibers (type I)) facilitating quantitative relationship between the two optical techniques. Wounded animals exposed to optimum dose of the red laser light displayed 5.70%, 5.90%, 1.73%, 1.43% and 1.09% fold increase in total collagen compared to un-illuminated control as reflected by the image analysis of Picro-Sirius stained histological sections. The microscopic images of the histological sections in RGB (red, green and blue) color space is firstly converted into HIS (hue, intensity and saturation) color scale through the “TissueQuant” software. The color score for each color in an image was decided based on how close the HIS components for the given color compared to the color of interest. Thus, color scores help to identify the color distribution in the positively stained regions of the histological specimens [22]. Color image analysis of Picro-Sirius red stained histological sections were performed previously [40], calculating the area of defined stained proteins (type I/III), ratios of collagen III:I, total collagen(sum of collagen type I and III) and collagen deposition over time (difference in total collagen between each time point) were used as a experimental endpoints to study the scarless fetal wound healing. Both “scores” [22] and “area” (pixel) [40], [44] have previously been used to quantify the presence of any particular substance in a given specimen. Our approach of expressing the total collagen (sum of type III and Type I) in percentage scores was adopted from the earlier reported work by Leila Cuttle and co-workers [40]. In the present study, Picro-Sirius red-polarization method, with combination of qualitative and quantitative assessments was explored, which provided a vital information on size, type, orientation, distribution and deposition of total collagen fiber that proved crucial for collagen monitoring following therapy. The findings of the present study corroborates with the earlier report work of da Silva and co-worker [42] involving collagen birefringence to indicate the presence of highly organized collagen bundles during skin repair following polarized red laser therapy.

Much similar to our present study, combination of two optical techniques i.e., autofluorescence and birefringence has also been previously utilized by Korol and co-workers [34] to examine the molecular changes in mature rat tail tendon. This study concluded that, LIF approach could be used as the future tool to investigate extracellular matrix changes during wound healing. The outcomes of the present *in vivo* autofluorescence have shown a significant difference in collagen synthesis between test and un-illuminated controls on day 5 post-wounding as compared to the corresponding histological image analysis. Although, the present study has utilized two sensitive techniques (autofluorescence and microscopy) for collagen assessment, autofluorescence has quite a few advantages over collagen birefringence microscopy. Firstly, it is rapid, and multiple fluorophores could be detected in a single spectrum, whereas birefringence in microscopy is a property of collagen and hence is restricted to only collagen excluding the measurement of other fluorophores in the tissues. Secondly, over the progression of the study, autofluorescence measurements were made in a non-contact mode, without disturbing the wound and allowing for non-invasive monitoring. As far as our understating, this is the first report of utilizing *in vivo* autofluorescence for an objective assessment of endogenous collagen during tissue repair following low dose of laser irradiation. Considering hemoglobin reabsorption as one of the limitations of the present technique, by combining it with appropriate imaging tool might be more suitable for its clinical application, avoiding the usage of repeated biopsy.

## Conclusions

In summary, the present study establishes the feasibility of *in vivo* autofluorescence for effective monitoring of diminutive changes in collagen levels during wound healing progression and suggested for its potential use as a non-invasive alternative tool over currently available *ex vivo* techniques. *In vivo* autofluorescence technique being non-invasive and objective may have clinical implications for collagen monitoring.
